# K-Alpha Calculator–Krippendorff's Alpha Calculator: A user-friendly tool for computing Krippendorff's Alpha inter-rater reliability coefficient

**DOI:** 10.1016/j.mex.2023.102545

**Published:** 2024-01-06

**Authors:** Giacomo Marzi, Marco Balzano, Davide Marchiori

**Affiliations:** a IMT School for Advanced Studies Lucca, Piazza San Francesco, 19, Lucca, Italy; b Department of Management, Ca’ Foscari University of Venice, 873 Cannaregio, S. Giobbe, Venice, Italy; c KTO Research Center, SKEMA Business School, 60 Rue Fedor M. Dostoevskij, Sophia Antipolis, France

**Keywords:** K-Alpha Calculator–Krippendorff's Alpha Calculator, Inter-rater, Inter-coder, Intercoder, Reliability, Krippendorff's Alpha, Krippendorff, Calculation, Calculator, Computation, Online Calculator, Human subjectivity

## Abstract

Krippendorff's Alpha is a measure for assessing inter-rater reliability, facilitating triangulated evaluations among multiple raters. Indeed, Krippendorff's Alpha is key in validating the dependability of human assessments, thereby reinforcing the robustness of human-based choices in contexts where interpretive variability could otherwise undermine the research outcomes. Despite its versatility across various data types, the procedure for computing this coefficient might limit its applicability for researchers unfamiliar with specialised statistical software. Addressing this issue, this paper introduces the “K-Alpha Calculator”—Krippendorff's Alpha Calculator—a freely accessible, user-friendly, web application available at https://www.k-alpha.org designed for easy computation of Krippendorff's Alpha. By offering a web interface without any software dependency, the K-Alpha Calculator seeks to promote the broader integration of such a reliability metric in research processes. We envisage that the tool's contribution could support researchers in enhancing methodological rigour, fostering conclusions are rooted in triangulated interpretations and assessments. The K-Alpha Calculator is both a computational tool and an educational resource, encouraging more researchers to include reliability measures in their studies.

• Development of the K-Alpha Calculator.The study centres on the introduction of a web-based application: the K-Alpha Calculator. The tool is developed to simplify the calculation of Krippendorff's Alpha without any required software dependency.
• User-friendly interface for diverse data types.The K-Alpha Calculator is characterised by its accessibility and user-friendliness, designed to simplify the procedures for computing the Krippendorff's Alpha. Its utility extends to various data types, which aligns with the intrinsic versatility of this coefficient.

## Graphical abstract

**Figure:**
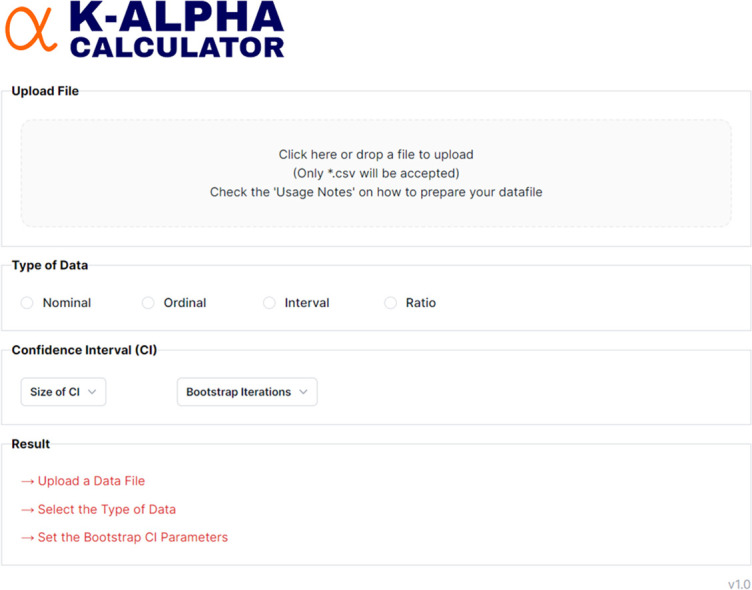

Specifications table

**Table:** 

Subject area:	Economics and Finance
More specific subject area:	Social Science, Multidisciplinary, Research Methods
Name of your method:	K-Alpha Calculator–Krippendorff's Alpha Calculator
Name and reference of original method:	Krippendorff's Alpha Inter-Rater Reliability Coefficient: Krippendorff, K. (2019). *Content Analysis: An Introduction to Its Methodology* (4th Ed.). SAGE Publications. https://doi.org/10.4135/9781071878781
Resource availability:	K-Alpha Calculator: https://www.k-alpha.org Source Code: https://github.com/davide-marchiori/k-alpha Permanent Repository for Archiving Purpose: https://doi.org/10.6084/m9.figshare.24847560

## Introduction

Across various fields, researchers are frequently confronted with the necessity of making subjective methodological choices [1]. Addressing this aspect, inter-rater reliability^1^ measures occupy a central position. Inter-rater reliability concerns the degree of agreement among evaluators (observers, raters, coders, analysts, and judges) when assessing the same set of items, ensuring ratings’ triangulation. Indeed, inter-rater reliability is key to establishing triangulation among multiple raters in different research contexts, especially when subjective judgments or categorisations are involved [1]. By evaluating inter-rater reliability, researchers can validate the rating protocols, minimising the threat that research outcomes are overly dependent on the evaluations of individual raters [1,2]. Various statistical measures can be employed to assess this reliability, with Cohen's Kappa [3], Fleiss’ Kappa [4], and Krippendorff's Alpha [1,2] being among the most widely used.

Inter-rater reliability provides confidence in the interpretative process and enhances the robustness of subsequent analyses and outcomes [5,6]. In this view, achieving high inter-rater reliability is evidence of the rating scheme's clarity and the raters’ rigorous training, improving outcomes’ robustness [5].

Indeed, when working with data, interpretation is inevitably filtered through human biases, perspectives, and judgments [1]. This inherent subjectivity becomes even more pronounced in collaborative research ventures or large-scale projects, where multiple raters might contribute to the interpretation process [1]. To build a shared ground for the research outcomes, it is thus necessary to rely on triangulated interpretation across the involved raters. This pursuit gives rise to the emphasis on inter-rater reliability measures [5].

Among the various statistical measures available for assessing inter-rater reliability, Krippendorff's Alpha stands out for its flexibility and adaptability to handle different levels of measurement and its capacity to manage missing data [1,2]. Originally developed by Klaus Krippendorff as an inter-rater reliability measure for the coding analysis of qualitative data where researchers dissect qualitative content—e.g., interviews, documental analysis, narrations or digital media—Krippendorff's Alpha plays a key role in reporting the agreement level among such interpretations [1,[7], [8], [9]]. As the categorisation of open-ended responses can be subject to varied interpretations [8], Krippendorff's Alpha ensures that these categorisations align with a triangulated interpretative framework (e.g., [9]).

Over the years, Krippendorff's Alpha has found applicability across diverse research domains for handling different data types—from binary and nominal to ordinal, interval, and ratio data [6], [7], [8]. For example, the application of Krippendorff's Alpha also extends to the domain of literature reviews, where multiple authors collaborate to identify relevant studies. During the preliminary stages of a literature review, researchers may conduct an initial search and subsequently discuss the subjective inclusion or exclusion of identified documents (e.g., [7]). This decision-making process is subject to varying interpretations regarding the relevance and content of the extracted studies. Thus, Krippendorff's Alpha can be instrumental in these scenarios, providing a statistical measure to estimate the agreement among the involved raters. Still, many other examples can be pointed out. Such examples of the applicability of Krippendorff's Alpha include observational studies, which often delve into the granular observation of behaviours in diverse contexts like classrooms, public spaces, or online platforms and can also have subjective interpretations of observed behaviours. Another field of applicability regards ethnographic research, which often employs coding or rating for elements like repeated behaviours, speech patterns, or cultural rituals [5].

However, the computational process for this coefficient, while rigorous, cannot be readily accessible for those not particularly familiar with existing on-topic statistical packages, software and available platforms. This could, in turn, limit the adoption of Krippendorff's Alpha inter-rater reliability coefficient into the research process, potentially reducing the research outcomes' robustness.

Addressing this gap, we introduce the K-Alpha Calculator—a free and user-friendly web-based application conceived to facilitate the calculation of Krippendorff's Alpha, available at https://www.k-alpha.org.

Beyond computational ease, this tool aims to enhance methodological rigour and allow researchers to minimise the threat of over-reliance on individual subjectivity. Next, we discuss the theoretical and methodological foundations of Krippendorff's Alpha, present the K-Alpha Calculator and its code, and offer a methodological template for accommodating its implementation in different studies.

## Theoretical and methodological foundations

Krippendorff's Alpha is a reliability coefficient developed to assess the agreement among observers, raters, coders, analysts, and judges that provide categorical, ordinal, interval, or ratio-level data [1]. The methodological underpinnings and procedural calculation of Krippendorff's Alpha are rooted in statistical formulations designed to provide a versatile reliability coefficient that spans across varying levels of measurement and accommodates any number of raters [1,2].

Central to the methodological implementation of Krippendorff's Alpha is the employment of coincidence matrices [1]. Such matrices detail the occurrences wherein multiple raters, among the possible rater combinations, allocate certain units of analysis to specific categories. In a coincidence matrix, each cell represents the frequency with which the multiple raters concur in their assignments for those units. The diagonal elements of this matrix represent instances of full agreement, whereas non-diagonal elements represent disagreements.

Moreover, Krippendorff's Alpha embeds elements of distance, or disagreement, between categories [1]. For categorical data, the distinction could be binary (e.g., 0= no; 1= yes) or in the form of categorisation (e.g. 1= Category 1; 2= Category 2; 3= Category 3). When one transitions to ordinal data, the disagreement takes the form of differences in rank order between categories (e.g., 1= Strongly disagree; 2= Disagree; 3= Neither agree nor disagree; 4= Agree; 5= Strongly agree). As one navigates further to interval or ratio-level data, squared differences between category assignments become instrumental in the calculus.

Methodologically, the distinction between observed and expected disagreements is central. The observed disagreement (*Do*) represents the disagreement derived from the rated data, whereas the expected disagreement (*De*) signifies the disagreement that could be expected under conditions of random coding.

The relationship between these two classes of disagreements is necessary to Krippendorff's Alpha, captured by the Eq. (1) presented by Krippendorff [1, p. 291]:

(1) $$\alpha =1-\frac{Do}{De}$$

To compute disagreements, researchers should engage in calculations tailored to the specific type of data in use. Such calculations involve aggregating distances between every pair of raters, normalising them, and eventually producing values of Do. For De, instead, it is assumed that raters operate independently and that their coding assignments are unrelated, relying on marginal frequencies to compute the expected distribution.

As a result, Krippendorff's Alpha can oscillate within the values of −1 to 1. A Krippendorff's Alpha value reaching the point of 1 indicates perfect reliability, representing a scenario with unanimity among raters [1]. In other words, a value of 1 translates to a situation where every rater uniformly assigns identical categories to each unit of content, manifesting full convergence in their evaluations. Conversely, when Krippendorff's Alpha drops to 0, it conveys that the reliability observed among the raters arises from pure randomness. A zero-value suggests that the coherence in raters' assignments bears no superior merit than if they were categorising content arbitrarily, lacking any structured guidelines or comprehensive understanding of the content [1].

A Krippendorff's Alpha below 0 implies an agreement scenario inferior to what might be anticipated from random assignments. Such a scenario emerges when raters are not merely in disagreement but might be making systematically opposed ratings. Such divergent assignments could stem from misunderstandings or severely contrasting interpretations of rating guidelines.

As a result, recalling Eq. (1), the quotient Do/De encapsulates the ratio of actual observed disagreement to the disagreement expected in the absence of any systematic coding effort. By subtracting this quotient from 1, the formula yields a reliability metric that spans from perfect agreement to pronounced systematic disagreement. The related mathematical explanation of Krippendorff's Alpha is extensively discussed by Krippendorff [1].

Overall, it is possible to delineate the following interpretations regarding the varying levels of Krippendorff's Alpha as suggested by Krippendorff [1, p. 356]:

1. **Alpha = 1**: Indicates perfect agreement among raters. It is the scenario where all raters have provided the exact same ratings for each item evaluated.
2. **Alpha ≥ 0.80**: This value is generally considered a satisfactory level of agreement, indicating a reliable rating. In many research contexts, a Krippendorff's Alpha equal to or above 0.80 is acceptable for drawing triangulated conclusions based on the rated data.
3. **Alpha [0.67 - 0.79]**: This range is often considered the lower bound for tentative conclusions. A Krippendorff's Alpha in this range suggests moderate agreement; thus, outcomes should be interpreted with concern, questioning the roots of such diverging ratings.
4. **Alpha < 0.67**: This is indicative of poor agreement among raters. Data with a Krippendorff's Alpha below this threshold are often deemed unreliable for drawing triangulated conclusions. It suggests that the raters are not applying the coding scheme consistently or that the scheme itself may be flawed.
5. **Alpha = 0:** Indicates no agreement among raters other than what would be expected by chance. It is similar to a random rating pattern.
6. **Alpha < 0**: A negative value of Krippendorff's Alpha indicates systematic disagreement among raters. This situation might arise in cases where raters are systematically inclined in opposite rating directions.

### Computational tools for Krippendorff's Alpha

Multiple tools have been proposed to compute Krippendorff's Alpha, each serving different research needs and technical proficiencies. They can be categorised into four major groups:

1. **Statistical Software Packages**: These packages, often part of feature-rich platforms, offer a wide range of statistical analyses, including the computation of Krippendorff's Alpha.
  • **R package “irr”**: integrated into the R environment, supports various inter-rater reliability and agreement coefficients [10].
  • **R package “icr”**: integrates the calculation of Krippendorff's Alpha in the R environment, supporting the bootstrap function [11].
  • **JASP** offers a free and open-source graphical interface for different statistical analyses. The range of various statistical tests includes inter-rater reliability analysis [12].
  • **Matlab add-on**: Matlab offers an add-on specifically for calculating Krippendorff's Alpha. This integration allows researchers to integrate inter-rater reliability analysis into Matlab's array of computational tools [13].
  • **KALPHA macro**: a macro for SPSS and SAS that computes Krippendorff's Alpha, allowing bootstrapping [2].
  • **Stata packages**: Different packages are available for Stata software to integrate inter-rater reliability analysis [14], [15], [16].
2. **Qualitative Analysis Software Packages**: These software packages are tailored for qualitative research, offering specific features for coding and analysing textual or multimedia data. Among their other functions, they allow the calculation of Krippendorff's Alpha, especially to evaluate the coding quality of the analysed data.
  • **NVIVO**: it supports a range of data types and is equipped with features to assess the inter-rater reliability of the coding performed inside the environment [17].
  • **MAXQDA**: this tool is designed for qualitative and mixed methods research and includes functionalities for assessing the coding reliability of data analysed with the package [18].
  • **Dedoose**: it offers a web-based platform for qualitative and mixed-methods research. Its capabilities include analysing text, video, and spreadsheet data alongside reliability analysis tools [19].
3. **Online Tools**: These are accessible, user-friendly platforms that compute Krippendorff's Alpha without the need for software installation.
  • **ReCal, ReCal OIR, ReCal3**: These online tools are specifically designed for calculating inter-rater reliability measures. They offer a simple interface with different calculation options [20,21].
4. **Python Libraries**: For researchers familiar with programming and data science, different Python libraries offer customisable solutions for computing Krippendorff's Alpha [22], [23], [24], [25]. A Ruby implementation of a Python library also exists [26].

As a result, in developing the K-Alpha Calculator, we aim to integrate the most spread functionalities inherent in the abovementioned software packages, while emphasising simplicity, accessibility, and independence from existing statistical packages.

Unlike comprehensive statistical packages or programming libraries, which often necessitate advanced technical skills, the K-Alpha Calculator is designed with a user-centric approach. Its interface and streamlined functionality make it accessible to researchers with varying statistical and computational expertise. Although easy to use, the calculator is equipped with capabilities such as bootstrapping and confidence interval calculations, which are functional to obtain more robust results.

## K-Alpha calculator software description

The underlying code of the K-Alpha Calculator executes the computational stepsas proposed by Krippendorff [1], validated with the examples from Krippendorff [1, p. 304]. It also incorporates the adjustments and considerations outlined in the bootstrapping method described by Hayes and Krippendorff [2].

Regarding the privacy of the uploaded data, the K-Alpha Calculator operates solely within the user's browser, guaranteeing that all data uploaded remains under the user's control. Its client-side functionality means that the K-Alpha Calculator does not retain, store, or transmit data to external servers, thereby adhering to data privacy standards. This approach avoids the risks associated with data storage and retention, establishing the K-Alpha Calculator as a secure tool for users concerned about data privacy.

### K-Alpha calculator algorithm

As described by Krippendorff [1] the computation of Krippendorff's Alpha measure rests on the definition of the *rates-by-units* matrix obtained by the reliability data matrix entered by users. It is worth noting that this approach allows for computing Krippendorff's Alpha for any of the metrics implemented (see below, Eqs. (3)–(6)), any number of observers, and in the presence of missing data.

The rates-by-units matrix displays the distribution of rates for each unit as follows (Table 1): where $n_{uc}$ is the number of rates c assigned to unit u; $n_{u.}=\sum_{c}n_{uc}$ is the number of rates assigned to unit u; $n_{.c}=\sum_{u|n_{u.}\geq 2}n_{uc}$ is the number of pairable values c occurring in the reliability data matrix (note that all units that have one or no rates are excluded); $n_{..}=\sum_{u|n_{u.}\geq 2}n_{u.}$ (again, excluding units with one or no rates).

**Table 1:** Matrix of the distribution of the rates.

Units	Rates					Totals
	1	…	c	k	…	
**1**	n_11_	…	n_1c_	n_1k_	…	**n_1_.**
**…**	…	…	…	…	…	
**2**	n_21_	…	n_2c_	n_2k_	…	**n_2_.**
**…**	…	…	…	…	…	**…**
**u**	n_u1_	…	n_uc_	n_uk_	…	**n_u_.**
**…**	…	…	…	…	…	**…**
	**n._1_**	**…**	**n._c_**	**n._k_**	**…**	**n..**

The Krippendorff's Alpha measure is computed via the following Eq. (2):

(2) $${}_{metric}\alpha =1-\;\frac{D_{o}}{D_{e}}=1-\left(n_{..}-1\right)\frac{\sum_{u}\frac{1}{n_{u.}-1}\sum_{c}n_{uc}\sum_{k>c}n_{uk}{}_{metric}\delta_{ck}^{2}}{\sum_{c}n_{.c}\sum_{k>c}n_{.k}{}_{metric}\delta_{ck}^{2}}$$

where the *metric* parameter indicates the scale on which the rates are measured and implies different forms of the *difference function* ${}_{metric}\delta_{ck}^{2}$.

Depending on whether the rates are measured on nominal (Eq. (3)), ordinal (Eq. (4)), interval (Eq. (5)), or ratio scale (Eq. (6)), the following difference functions are:

(3) $${}_{nominal}\delta_{ck}^{2}=\left\{ \begin{array}{c} 0\;iff\;c=k\\ 1\;iff\;c\neq k \end{array}\right.$$

(4) $${}_{ordinal}\delta_{ck}^{2}={\left(\sum_{g=c}^{g=k}n_{.g}-\frac{n_{.c}+n_{.k}}{2}\right)}^{2}$$

(5) $${}_{interval}\delta_{ck}^{2}={\left(c-k\right)}^{2}$$

(6) $${}_{ratio}\delta_{ck}^{2}={\left(\frac{c-k}{c+k}\right)}^{2}$$

Confidence intervals for the computed Krippendorff's Alpha measure are obtained through a bootstrap procedure [2]. Firstly, the user selects the level for the confidence interval and the size of the population of bootstrap reliability data matrices to be produced. Based on these inputs, the software creates a population of data matrices, each with as many rows (i.e., units) as the original one, by randomly drawing (with repetition) rows of the initial reliability data matrix. Then, the Krippendorff's Alpha value for each bootstrap data matrix is computed and stored. Finally, the (symmetric) confidence interval is determined based on the opportune quantiles of the obtained distribution of Krippendorff's Alpha measures.

### K-Alpha usage guide

**Fig. 1:**
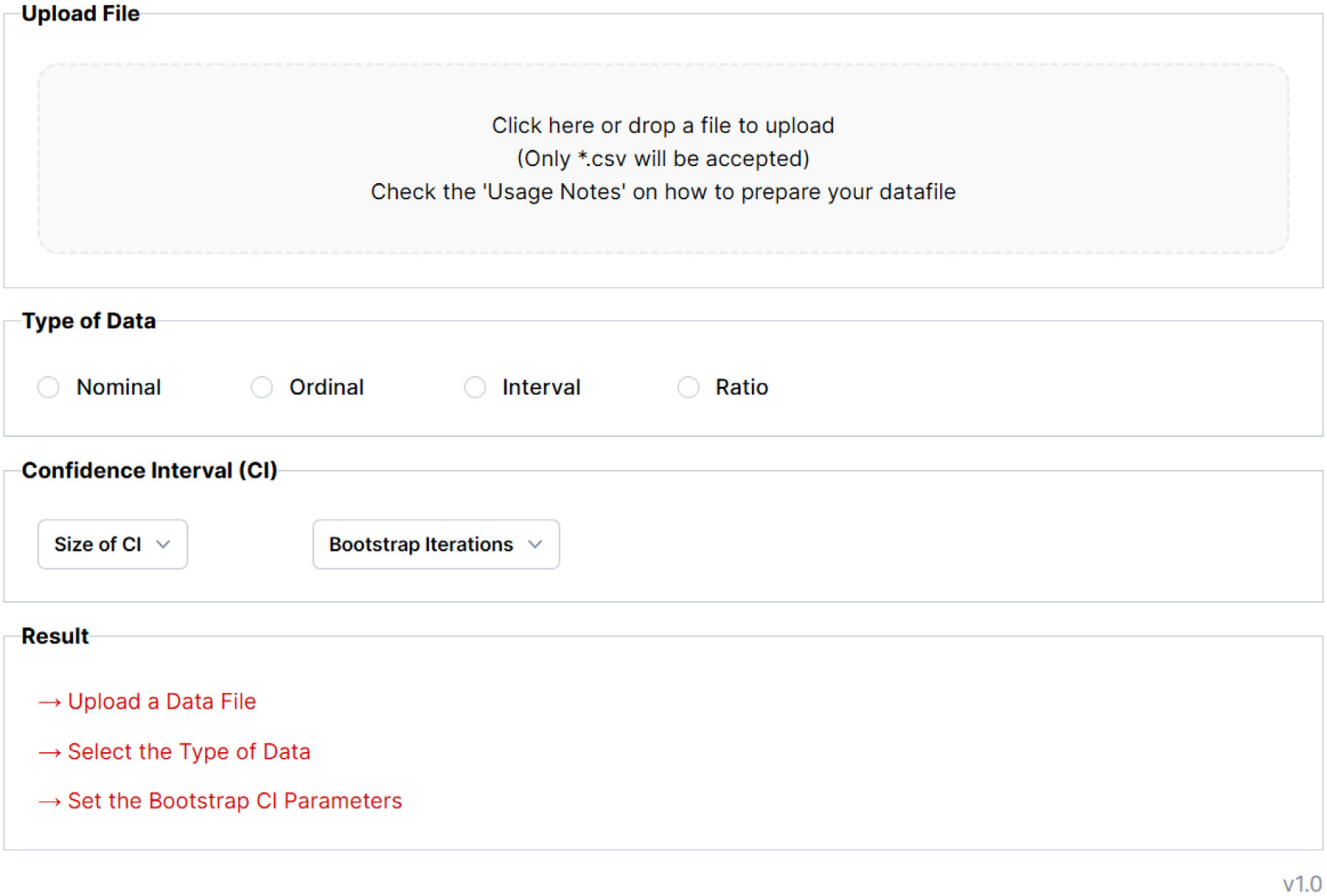
K-Alpha Calculator user interface.

Below, we aim to provide guidance on the user interface's key components, which can be found at https://www.k-alpha.org, and reported in Fig. 1. The open-source code can be found on GitHub at: https://github.com/davide-marchiori/k-alpha

The K-Alpha Calculator operates using a three-step approach. Firstly, users upload a properly formatted data file. Subsequently, they specify the data type; there is an option to select confidence intervals and the size of the bootstrap. Finally, users can view the resulting Krippendorff's Alpha value. The three steps are detailed as follows:

*1. Data Upload:* K-Alpha Calculator requires users to upload their data in a .csv format. The upload section provides an action point where users can either click to browse and choose a .csv file or directly drag and drop it into the designated area. The .csv file must comply with the following characteristics:

**File Type:**

• Required format: .csv (Comma-Separated Values).
• Delimiter: Comma (,) or semicolons (;). Tab or other delimiters are not permitted.
• Size limit: 500 KB; only one data file can be uploaded at a time.
• The file should not contain any headers, footers, or empty spaces.

**Layout:**

• Organised in matrix format.
• Rows: Represent individual items rated
• Columns: Indicate raters.
• Rate Representation: Numerical values, no decimals.
• 'NA' (without quotation marks): Denotes missing values. No empty spaces are allowed.
• Not include the names of the items or the names of raters in the data file.

**Content:**

• Matrix cell: Contains the rater-assigned rate to an item.
• Data Types: Nominal, ordinal, interval, or ratio.

Once the data file is uploaded, the K-Alpha Calculator reads the file and provides key information to be checked by authors. Information includes the file size, date and time of the last modification, number of raters identified, number of rated items identified, and number of missing values identified.

*2. Data Type Specification:* Users should delineate the nature of the data upon uploading the data file. This specification can be selected from the following options via a radio button: Nominal, Ordinal, Interval, and Ratio. The data type selection significantly influences the computational method employed for calculating Krippendorff's Alpha.

Users have the option to calculate confidence intervals (CI). A dropdown menu is provided to simplify this process, offering three confidence interval levels: 90%, 95%, and 99%. Additionally, users are required to select the number of bootstrap iterations from the available options of 200, 400, 600, or 1000. It is important to note that the bootstrap computation is executed using the user's computational resources rather than being performed by the web application.

*3. Result Display:* The calculator processes the input data and presents Krippendorff's Alpha value in the output section. For a comprehensive analysis, this section also exhibits the minimum and maximum rates alongside the count of valid rates rated more than once. The Krippendorff's Alpha value is also displayed, with the specified data type indicated in brackets. If a confidence interval is selected, the output includes its upper and lower bounds, providing a more nuanced understanding of the reliability estimate.

## Illustrative examples

Consider a scenario involving 5 items, 4 raters, and 3 categories represented by the values '1', '2', '3'(nominal data). The corresponding data file should consist of 5 rows, each representing an item, and 4 columns, each corresponding to a rater. The values in the cells represent the rates assigned by the raters. An illustrative example of such a data arrangement is presented below:

**Table:** 

1,1,1,1 2,2,3,2 3,3,3,3 3,3,3,3 2,2,2,2

### Missing values

In cases where a rater has not assigned a rate to an item, the cell should be marked with 'NA' (without quotation marks). For instance, if the third rater did not assign a rate to the first item and the second rater did not assign a rate to the fourth item, the data file should be formatted as follows:

**Table:** 

1,1,NA,1 2,2,3,2 3,3,3,3 3,NA,3,3 2,2,2,2

### Exempary calculation

**Fig. 2:**
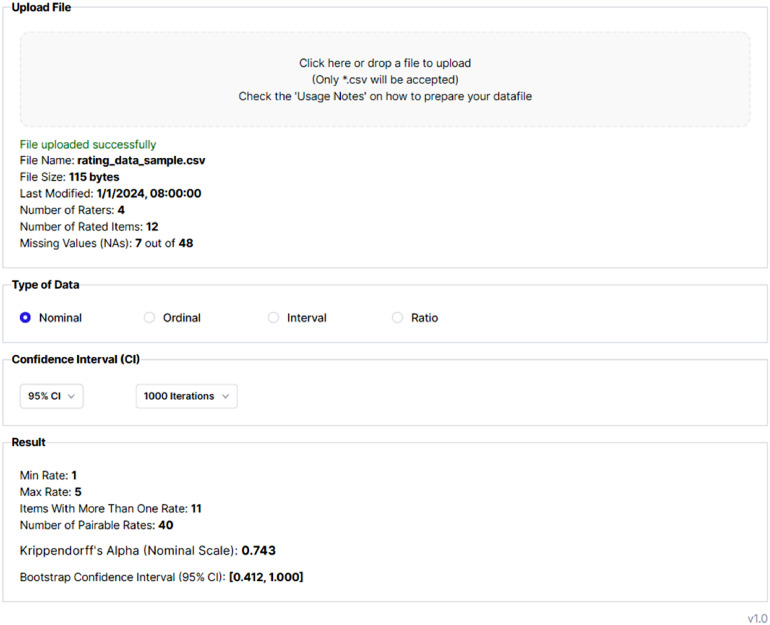
K-Alpha Calculator exemplary calculation.

Fig. 2 illustrates the interface of the K-Alpha calculator for calculating Krippendorff's Alpha with 4 raters, 12 rated items, 7 missing values, 5 different categories (nominal data). The data presented in this figure are obtained from the examples provided by Krippendorff [1, p. 304] as follows:

**Table:** 

1,1,NA,1 2,2,3,2 3,3,3,3 3,3,3,3 2,2,2,2 1,2,3,4 4,4,4,4 1,1,2,1 2,2,2,2 NA,5,5,5 NA,NA,1,1 NA,3,NA,NA

As depicted in Fig. 2, the calculated Krippendorff's Alpha is 0.743. The raters' ratings range from a minimum of 1 to a maximum of 5. Notably, one item received only one rating, accompanied by three 'NA' codes, resulting in 11 valid items for calculation, with 40 pairable ratings. The confidence intervals, determined at a 95% level with 1000 bootstrap iterations, yield lower and upper bounds of 0.412 and 1.000, respectively.

## Methodological template

In both qualitative and quantitative research domains, reporting the reliability of data coding accurately and effectively presents a significant challenge. To facilitate this, we introduce a methodological template specifically crafted to assist researchers in articulating using Krippendorff's Alpha within their studies (Box 1). This template guides the description and justification of employing Krippendorff's Alpha as a reliability measure, aiding in the category creation process and subsequent reporting of results obtained via the K-Alpha Calculator.

The template (Box 1) features sections in *italics,* enclosed in [brackets], which are designated for user customisation. This structured approach aims to streamline the methodological narration, making it rigorous and tailored to the specific needs of different research contexts.

Box 1 **Methodological template**

A group of *[Number]* raters with backgrounds in *[Pertinent fields]* took part in this study *[Here, the raters themselves may be the authors of the study].* Before coding, they underwent a training session that included familiarising them with the coding scheme, practicing coding exercises, and discussing to clarify any ambiguities.

The coding scheme was developed based on *[Here, the authors should delineate the theoretical framework or scholarly literature that informed the development of their coding scheme].* It consisted of *[Number]* categories, each with a defined set of attributes *[Here, authors could describe in detail the characteristics of each category].*

Krippendorff's Alpha was employed to assess the inter-rater reliability of the rating scheme (Krippendorff, 2019). This statistical measure is particularly suited for studies with multiple raters and different levels of measurement. It is a robust measure that accommodates any number of raters, sample sizes, and missing data, making it appropriate for numerous study designs (Krippendorff, 2019).

In the present case, each rater independently provided ratings. The rated data were then input into the web-based statistical package K-Alpha Calculator (Marzi et al., 2024). The analysis provided a reliability coefficient for the coding scheme, indicating the extent of agreement among raters beyond chance. The resulting Krippendorff's Alpha coefficient is *[Insert the Krippendorff's Alpha value calculated with* K-Alpha *Calculator, eventually including the Confidence Intervals (CI)],* recalling that the threshold for a satisfactory level of this coefficient is 0.80, as suggested by Krippendorff (2019).

**References for the Methodological Template**

Krippendorff, K. (2019). *Content Analysis: An Introduction to Its Methodology* (4th Ed.). SAGE Publications. https://doi.org/10.4135/9781071878781

Marzi, G., Balzano, M., & Marchiori, D. (2024). K-Alpha Calculator —Krippendorff's Alpha Calculator: A User-Friendly Tool for Computing Krippendorff's Alpha Inter-rater Reliability Coefficient. *MethodsX,* 102545. https://doi.org/10.1016/j.mex.2023.102545

Alt-text: Unlabelled box

## Conclusion

Reliable and triangulated data ratings in different research domains are increasingly demanded to enhance methodological rigour [1,[2], [3], [4], [5], [6], [7], [8]]. In this perspective, inter-rater reliability is key for promoting the validity and robustness of research outcomes. Krippendorff's Alpha stands out for its versatile applicability across diverse data types and research contexts among the various statistical measures employed for this purpose. However, procedures for computing this coefficient might limit its applicability for researchers unfamiliar with specialised statistical software.

To overcome this issue, we introduce the K-Alpha Calculator–a free, web-based application specifically designed to simplify the computation of the Krippendorff's Alpha. This software offers a user-friendly interface, rendering it accessible even for individuals with limited statistical expertise. The K-Alpha Calculator aims to democratise access to this key measure of reliability. Moreover, the K-Alpha Calculator extends its utility by incorporating advanced features like bootstrapping and confidence interval calculations. These additions improve the robustness of the results, further introducing trust in the research outcomes. In conclusion, introducing the K-Alpha Calculator holds implications for the academic community, enhancing the rigour of research outcomes by making a critical statistical tool more accessible, contributing to improving research methods by providing an easy-to-use platform for reliability calculations, and serving as an educational resource, potentially stimulating more researchers to incorporate reliability measures in their studies.

## Ethical statement

As the authors and maintainers of the K-Alpha Calculator, we are committed to preserving high standards of ethical conduct in data management and user privacy. We assure users that all data input into the K-Alpha Calculator remains exclusively within the user's domain, as the calculator operates entirely on the client side. This means it functions solely within the user's browser environment, guaranteeing that no data is retained, stored, or transmitted to external servers or storage systems.

## CRediT authorship contribution statement

**Giacomo Marzi:** Conceptualization, Methodology, Writing – original draft, Writing – review & editing, Supervision, Project administration. **Marco Balzano:** Conceptualization, Methodology, Writing – original draft, Writing – review & editing. **Davide Marchiori:** Writing – review & editing, Software, Validation.

## Declaration of competing interest

The authors declare that they have no known competing financial interests or personal relationships that could have appeared to influence the work reported in this paper.

## Data Availability

No data was used for the research described in the article.

## Footnotes

1 Especially in the domain of qualitative studies, where evaluators are involved in the coding process of data, inter-rater reliability is often reported as 'inter-coder reliability'. In this paper, the term 'inter-rater reliability' is used in its broadest sense, encompassing all types of evaluators mentioned (observers, raters, coders, analysts, and judges). This inclusive approach is adopted to portray a comprehensive assessment of agreement levels across different evaluation roles.
